# The First Female Dry Immersion (NAIAD-2020): Design and Specifics of a 3-Day Study

**DOI:** 10.3389/fphys.2021.661959

**Published:** 2021-06-14

**Authors:** Elena Tomilovskaya, Liubov Amirova, Inna Nosikova, Ilya Rukavishnikov, Roman Chernogorov, Svetlana Lebedeva, Alina Saveko, Ivan Ermakov, Ivan Ponomarev, Inna Zelenskaya, Tatiana Shigueva, Nikita Shishkin, Vladimir Kitov, Alexandra Riabova, Vitaly Brykov, Nelly Abu Sheli, Galina Vassilieva, Oleg Orlov

**Affiliations:** Russian Federation State Scientific Center – Institute of Biomedical Problems of the Russian Academy of Sciences, Moscow, Russia

**Keywords:** Dry immersion, women, ground-based model of microgravity, supportlessness, NAIAD-2020

## Abstract

This article describes procedures and some results of the first study of females undergoing 3-day Dry Immersion. The experiment “NAIAD-2020” was carried out at the Institute of Biomedical Problems (Moscow, Russia) with the participation of six healthy women volunteers (age 30.17 ± 5.5 years, height 1.66 ± 0.1 m, weight 62.05 ± 8.4 kg, BMI 22.39 ± 2.2 kg/m^2^) with a natural menstrual cycle. During the study, a standard protocol was used, the same as for men, with a minimum period of time spent outside the immersion bath. Before, during and after Immersion, 22 experiments were carried out aimed at studying the neurophysiological, functional, metabolic and psychophysiological functions of the body, the results of which will be presented in future publications. The total time outside the bath for women did not exceed that for men. Systolic and diastolic pressure did not significantly change during the immersion. In the first 24 h after the end of the immersion, heart rate was significantly higher than the background values [*F*(4,20) = 14.67; *P* < 0.0001]. Changes in body temperature and water balance were consistent with the patterns found in men. No significant changes in height and weight were found during immersion. All women reported general discomfort and pain in the abdomen and back. The results of this study did not find significant risks to women’s health and showed the feasibility of using this model of the effects of space flight in women of reproductive age.

## Introduction

As the participation of women in space flight missions becomes more and more regular, it is of importance to study women’s health under the influence of space flight factors (Mark et al., 2014). In particular, previous studies have shown that women are more susceptible to orthostatic stress (Platts et al., 2014), space motion sickness (Reschke et al., 2014), and radiation (Kennedy et al., 2014). Despite the fact that gender differences should be taken into account during the preparation for long-term missions, this issue has not been fully studied, in particular, due to a small number of female cosmonauts/astronauts.

Therefore, the attention of researchers is focused on the study of functions of the female body not only in a real space flight, but also in model conditions. The most popular model for reproducing space flight factors, head down bed rest (HDBR), has also been used for women. Such studies were initiated even before regular missions (Pace et al., 1976) and, in some cases, were aimed at studying cardiovascular responses to HDBR. It was shown that, the production of noradrenaline in response to orthostatic stress after 7-day HDBR in women was reduced (Millet et al., 2001), as was the tolerance time of the orthostatic test (Custaud et al., 2002). The most extensive studies of the female body under HDBR conditions with an emphasis on the cardiovascular system were carried out at MEDES (Toulouse, France) in 2005. Under conditions of 60-day HDBR in 24 female volunteers, physical exercise demonstrated a prophylactic effect compared with the group taking protein supplements (*n* = 8) and the control group (*n* = 8) (Demiot et al., 2007; Hughson et al., 2007; Beller et al., 2011; Lee et al., 2014; Holt et al., 2016). The longest study of women’s exposure to HDBR was carried out at the IBMP (Moscow, Russia) in 1994. During the 120-day exposure, the effects of HDBR *per se* (*n* = 4) and HDBR combined with physical exercise (*n* = 4) on the state of cardiovascular (Maillet et al., 2000) and neuromuscular (Koryak, 1998) systems, as well as on insular activity (Afonin, 2018), bone mineral density (Oganov et al., 1997), and cholesterol metabolism (Mukhamedieva et al., 2018) were studied.

Despite the popularity of HDBR as a model, it requires a long exposure time, which can lead to additional difficulties. Less demanding in this regard is the model of Dry Immersion which reproduces the support unloading conditions. This model has been successfully used in men for over 50 years (Shul’zhenko and Vill-Villiams, 1976). The main acting factors of this model are hydrostatic compression, as well as physical and support unloading. The basic difference between DI and HDBR models is the level of support (weightbearing) decrease. Being submerged in the column of liquid, close in density to the human body, and uniformly flowing around it, the subject is almost in support unloading conditions. This condition is similar to weightlessness, with water hydrostatic pressure distributed equally over the body surface (Grigor’ev et al., 2004; Navasiolava et al., 2011). Dry immersion promotes rapid gravitational deconditioning for somatosensor, cardiovascular and other systems (Navasiolava et al., 2011; Tomilovskaya et al., 2019; Amirova et al., 2020b). Dry immersion, as well as HDBR, is accompanied by central hypervolemia, however, there is also evidence that DI has a more powerful effect on the cardiovascular system than −8° HDBR does (Krupina et al., 1982). It has also been shown that in a short time, within 3–7 days, there is a change in the sensorimotor functions: a decrease in accuracy of movement control, decline in the accuracy of visual tracking, and an increased sensitivity to vestibular signals. The physiological aspects of these models, obtained from participation of male volunteers, are described in details in the review articles (Pavy-Le Traon et al., 2007; Navasiolava et al., 2011; Watenpaugh, 2016; Tomilovskaya et al., 2019; Pandiarajan and Hargens, 2020). However, studies involving this model in women have never been conducted, in part, due to a number of hygienic and physiological difficulties.

The aim of this study was to conduct a short-term 3-day immersion, as well as to analyze the tolerance to exposure and describe the medical risks in a group of women of reproductive age at these conditions.

## Materials and Methods

### Participants

The study was carried out with the participation of six female volunteers of reproductive age with a natural menstrual cycle (MC), without identified acute and chronic diseases and involved only one group of women. The subjects had never been subjected to microgravity model studies. The detailed characteristics of research participants are provided in Table 1.

**TABLE 1:** Age, height, weight, and body mass index (BMI) of participants.

**Number of participants**	**Age, years**	**Height, m**	**Weight, kg**	**BMI, kg/m^2^**
1	34	1.781	64.5	20.3
2	39	1.545	46.8	19.6
3	26	1.64	58.5	21.8
4	28	1.719	68.3	23.1
5	24	1.616	64.7	24.8
6	30	1.675	69.5	24.8
Mean	30.17	1.66	62.05	22.39
SD	5.5	0.1	8.4	2.2

### Bioethics and Informed Consent

The conducted studies were approved by the Bioethical Commission of the Institute of Biomedical Problems of RAS (Protocol No. 544 of July 16, 2020) and fully complied with the principles of the 1964 Declaration of Helsinki.

Each study participant voluntarily signed an informed consent after having the potential risks, benefits and nature of the upcoming study explained to her.

### Design of the Study

**FIGURE 1:**
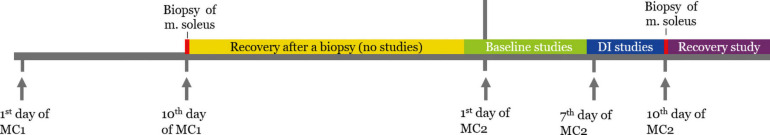
Design of the study of female 3-day Dry Immersion. MC, menstrual cycle; DI, Dry Immersion.

The experiment “NAIAD-2020” was carried out at the Institute of Biomedical Problems of RAS from September 7 to November 30, 2020. The women’s immersion experiment took into account their specific features associated with gender. Each participant was involved in the study for as long as two menstrual cycles (MCs) (Figure 1). The average duration of the MC was 28.4 ± 2.8 days. It is worth noting that in the first days of the cycle (the first 2–3 days), in some cases, it was necessary to introduce restrictions in the load methods (study #6 from Supplementary Table 1 was carried out no earlier than on day 3 of MC). In two test subjects, menstruation lasted for 7 days and ended on day 1 of DI; other cases, menstruation ended before the start of DI. On the 10th day (during this period, there was a reduced concentration of estradiol in the blood) of the first cycle, a biopsy sample of the soleus muscle of the shin of the left leg was taken. Also, in the period from 11th to 13th days of the MC, gynecological smears were taken, after which no experimental studies were carried out until the end of this cycle. The start of baseline studies was at the first day of the second cycle. However, due to the natural mobility of the cycle, baseline studies could begin several days before the first day of MC1. The start of immersion for all participants was at the 7th day of the cycle, and its completion – at the 10th day. Recovery period studies continued for another 7 days after the completion of the immersion exposure (Supplementary Table 1).

### Tests

The list of tests taken before, during, and after 3-day DI is presented in Supplementary Table 1. For the implementation of some techniques, the subject was raised from the immersion bath for a short time; during such tests, she was in the supine position.

Biopsy samples were taken twice using the Bergström needle biopsy technique with aspiration under local anesthesia (2 ml of 2% lidocaine) from the soleus muscle of the shin of the left leg. Venous blood sampling for various tests was performed four times: once before the start of DI, after 24 and 72 h of immersion and on the 6th (or 7th) day of the recovery period. The total volume of venous blood collected during the entire experiment was 115 ml per subject.

### Immersion Conditions

According to the conditions of the experiment, the subjects were immersed for 3 days. They were allowed to leave the immersion bath for some examinations and hygiene procedures. The average time spent by the subjects outside the immersion bath was 28.2 ± 2.8 min on the first day, 22.1 ± 0.9 and 18.1 ± 0.6 min on the second and third days, respectively (Supplementary Figure 1). Approximately 2/3 of the total time outside the immersion bath the subjects spent in the supine position. In the immersion bath, the subjects’ movements were moderately restricted; physical exercises were not performed. The water temperature was maintained at 32.5 ± 2°C. Every evening, the subjects were raised from the bath for an average of 15–20 min to carry out hygiene procedures, most of which were performed in the supine position. The subjects were under 24 h surveillance, including monitoring both main vital parameters and the working condition of immersion baths and other technical equipment. The day before the start of the immersion (B-1), every day of the immersion (DI1–DI3), as well as the day after its completion (R + 0), a comprehensive medical examination was performed three times a day (15:00, 21:00, and 8:00); the list of examinations is provided in Supplementary Table 2. During the time free from treatments and tests, the subjects had the opportunity to read, work on a laptop, watch TV, talk on the phone, etc.

**FIGURE 2:**
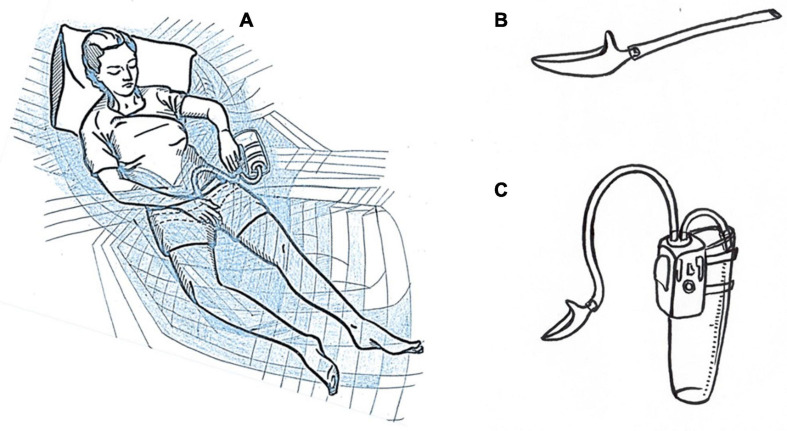
Scheme of a universal portable device for mechanical urine collection in women **(A)**. Urine collection nozzle **(B)**. Fully assembled device **(C)**.

To solve the problem of urine collection, subjects used a special urine collector (Lebedeva, 2020) which allows urination while lying in an immersion bath (Figure 2A). The device consists of a miniature urine collection funnel (Figure 2B) connected with a flexible silicone tubing to the apparatus (Figure 2C) which comprises a diaphragm pump, control boards, variable resistor and battery. Via a second silicone tubing, the pump was connected to a container for storing and transporting urine. This method of urine collection allows researchers and physicians to conduct adequate volumetric measurements and further clinical analysis, excluding contamination with vaginal secretions. Urination was possible in any position using this device. In this experiment in most cases, the device was used in support unloading conditions, but sometimes solid support was required, as described in section “Medical Traits and Risks.” A pillow under the back and shoulders was commonly used.

The day before the start of the experiment, during the entire immersion period and the day after its completion, the subjects were on a controlled diet with fluid intake *ad libitum*. A standard menu was developed with three meals a day, taking into account the taste preferences of the participants. The calorie content of the diet was calculated according to the formulas used for determining the energy value of the food rations for the ISS crew members. The recommended daily caloric intake was 2,315 ± 117 kcal/day for women under 30 years old, 2,224 ± 166 kcal/day for women over 30 years old, balanced in basic nutrients. Mineral composition of the diet was not calculated. During the day, a few snacks were allowed (fresh and dried fruits, cookies, candies). Indigestible, gas-forming, spicy and high fat food products were excluded from the diet. The use of alcohol, caffeine-containing products, nicotine, cannabinoids, and exogenous opiates was completely prohibited throughout the entire study period. To maintain a comfortable psychophysiological state during the immersion period, subjects were allowed some deviations from the proposed menu that were recorded in the food diary.

### Statistical Analysis

Statistical analysis was performed using GraphPad Prism 8.3 software. The applied criteria are given in Supplementary Table 2. The established significance level was *p* < 0.05.

## Results

### Blood Pressure and Heart Rate

Systolic blood pressure (SBP) values before the start of immersion were 109.3 ± 4.2 mm Hg in the evening and 110.5 ± 4.5 mm Hg in the morning (Supplementary Figure 2A). During immersion, blood pressure lowered (on average by 2.8 mm Hg in the evening and by 6.8 mm Hg in the morning), but no significant differences were found.

Diastolic blood pressure (DBP) values before immersion were 70.3 ± 3.4 mm Hg and 75.6 ± 4.8 mm Hg in the evening and in the morning, respectively (Supplementary Figure 2B). During immersion, DBP was slightly lower than the initial values, returning to them after the completion of the DI.

The initial values of heart rate (HR) of the subjects on average were 70 beats per minute, slightly decreasing during the exposure (Supplementary Figure 2C). However, after coming out of immersion, a significant increase in HR was recorded by 18 and 14 beats per minute in the evening and in the morning, respectively [*F*(4,20) = 14.67; *P* < 0.0001].

It is worth noting that none of the participants, after coming out of immersion, had a critical decrease in blood pressure with the development of fainting.

### Monitoring of Body Temperature and Environment

**FIGURE 3:**
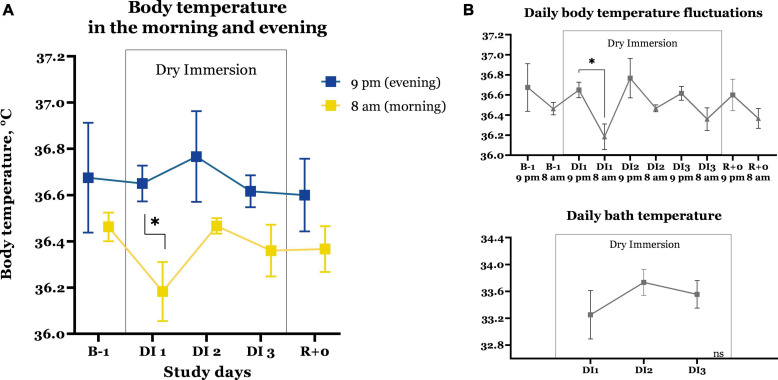
Changes in body temperature of the subjects in the morning and in the evening **(A)**, daily fluctuations **(B)**, and water temperature in the immersion bath **(C)**. Mean ± SEM. * -significant difference, *P* < 0.05.

Changes in the morning and evening body temperature, as well as its daily fluctuations are shown in Figure 3. Before immersion, the body temperature of the subjects in the morning was 36.46 ± 0.06°C. Almost 24 h after staying in immersion, the body temperature of the subjects was significantly lower [*F*(1,5) = 16.73; *P* = 0.0463], which can be explained by adaptive fluctuations of thermoregulation under immersion conditions (Amirova et al., 2016). In the subsequent days of immersion and during the period of recovery, no spikes in values were observed. The body temperature of the subjects in the evening was higher, as expected, ranging from 36.6 to 36.76°C; there was no correlation with staying in the immersion bath.

The temperature in the immersion bath on average for the group of subjects was stable and ranged from 33.2 ± 0.4°C to 33.5 ± 0.2°C (the difference was not statistically significant).

### Water Balance

**FIGURE 4:**
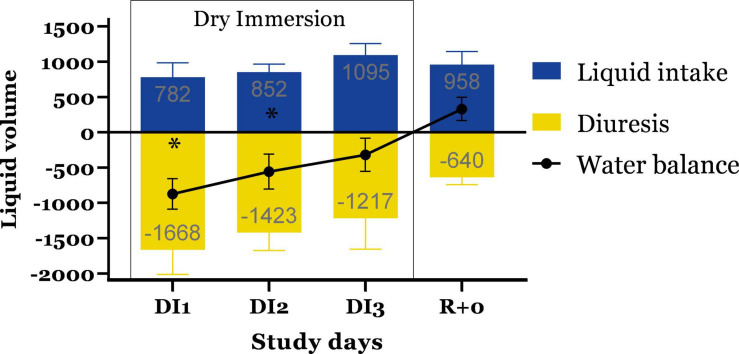
Daily volume of consumed and discharged fluid, as well as the water balance. Mean ± SEM. * <0.05 vs. R + 0.

During 3 days of immersion, subjects’ water balance remained negative vs. R + 0 [*F*(4,25) = 4.183; *P* = 0.0099] due to reduced fluid intake and increased diuresis (Figure 4). After completion of immersion, the water volume of the excreted liquid decreased and water balance became positive +318.3 ± 164.5 ml.

### Body Height and Weight

**FIGURE 5:**
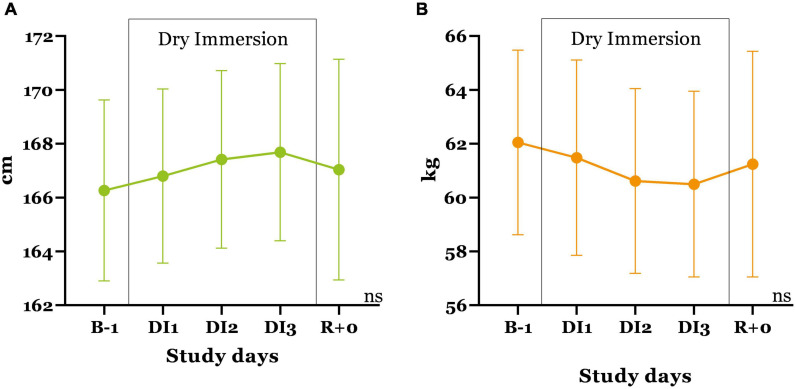
Changes in the height **(A)** and body weight **(B)** of the subjects during the 3-day immersion exposure. Mean ± SEM.

The initial height of the subjects was 166.6 ± 3.3 cm (Figure 5). During immersion, a progressive increase in the height of the subjects was recorded, comprising on average 1.4 cm per group. Body weight before the immersion was 62.0 ± 3.4 kg; by the third day, it decreased to 60.1 ± 3.4 kg. In the first 12 h out of immersion, height and weight showed a recovery trend.

### Discomfort

The process of adaptation to the conditions of simulated microgravity is associated with the reaction of the organism to compensate for external influences, which may be accompanied by symptoms of discomfort, including the pain syndrome.

On the day before the immersion, two subjects noted general discomfort of 1–2 points (Supplementary Figure 3A). On the first day of immersion, five out of six subjects reported discomfort; most rated it at 3 points. However, one participant rated the level of general discomfort at 7–8 points. In the subsequent days of immersion, the level of discomfort decreased. However, the general discomfort remained in some participants (up to 3 points) after the completion of the immersion.

Five participants reported back pain under DI conditions (Supplementary Figure 3B). Two participants reported pronounced discomfort with appreciable back pain (up to a maximum of 3–5 points) on the first and second days of DI. On the first day after the completion of the immersion, one subject rated back pain at 1 point.

All participants reported abdominal discomfort of the bloating type and false urges to defecate under DI conditions throughout the immersion (Supplementary Figure 3C). The most intense sensations (4–6 points) were noted in two participants in the first 2 days of immersion. At the same time, extraction from the immersion bath relieved the discomfort. Also, when a subject was extracted from the immersion bath in the case of pronounced discomfort and the urge to defecate, the act of defecation did not occur and the urge turned out to be false. In some cases, stool retention up to 3 days was observed, after which the act of defecation returned to normal and false urges did not appear. After the completion of the immersion, the subjects did not note any discomfort or pain in the abdomen.

### Medical Traits and Risks

During Dry Immersion experiments in women, we noted a distinct trait that was not observed (or expressed to a much lesser extent) in men (Tomilovskaya et al., 2020). All women in the group noted some problems with urination expressed in the urge to urinate without the possibility of its fulfillment. In most cases, this problem was solved by completely relaxing the body, choosing the correct posture and the size of the receiving funnel. At the beginning in some cases it was necessary to raise the solid platform to ease urination (the subject remained in the supine position). However, in one case the subject had to be extracted from the immersion bath to toilet for the act of urination. Two out of six subjects had symptoms of “insensitive bladder” manifested by involuntary diuresis. After 2 days of immersion exposure, four subjects did not have dysuric phenomena, although in one case out of six the phenomenon of dysuria was present throughout the entire immersion and urination returned to normal on the second day after the completion of the experiment.

On the first day of immersion, one subject had spontaneous vomiting which presumably appeared as a result of mental overstrain. An attack of vomiting was stopped by using medication, after which urges to vomit did not appear.

The condition of the subjects’ skin did not change, which can most likely be explained by both the short duration of exposure and the small size of the group.

The menstrual cycle after completion of participation in the experiment remained intact for all subjects.

## Discussion

### Main Findings

In this study, we showed the possibility of participation of female volunteers in experiments under conditions of Dry Immersion; no significant contraindications were found during the 3-day exposure. We noted some medically distinct traits related to female physiology which required observation during the experiment.

### Comparison vs. Real Spaceflight

The vast majority of physiological data in space flights have been obtained with men. Often, original researches provide averages of participants of both sexes, with no focus on gender differences, making it difficult to analyze the literature. Nevertheless, we found that the decrease in body weight in women in the first month of the flight is 2–5% of the pre-flight values (Zwart Sara et al., 2014). The decrease in body weight in our case was 7% for 3 days of exposure, which is most likely due to hypovolemia (de Abreu et al., 2017).

Survey results show that approximately half of all astronauts (58% of whom are female astronauts) report back pain during the early stages of space flight (Sayson and Hargens, 2008; Kerstman et al., 2012). Back pain was most often reported on the first and second days of SF, and 75% felt it at night. In our study, the frequency and time of day of reports of back pain matched the flight data (Kerstman et al., 2012). Previous research suggests that the phenomenon of back pain may be associated with the height increase (Rukavishnikov et al., 2017). In this regard, it is interesting to note that women neither significantly increased their height, nor experienced significant discomfort in the lumbar region, which requires further research.

The generalized experience shows that women have experienced a higher incidence of urinary tract infections in space (Mark et al., 2014). Possible explanations for this sex difference include women’s urethral anatomy, adjustment to voiding in microgravity, a higher incidence of dysuria, and a higher incidence of catheterization (National Aeronautics and Space Administration (NASA), 2014; Ronca et al., 2014). In our study, we noted the phenomenon of dysuria, but the incidents were resolved safely without catheterization. Before the experiment, all subjects successfully tested the urine collector for the DI, therefore the phenomenon of dysuria under the conditions of immersion exposure was unexpected both for the researchers and for the participants themselves. Also, it should be noted that during immersion women showed a more pronounced psychological discomfort during urination than men did. However, it remains unclear whether the discomfort was a cause or, a consequence of dysuria. Only healthy women without pathology of the genitourinary system were selected for participation in the experiment, therefore, with a high degree of probability, it is possible to exclude neurogenic bladder dysfunction, expressed in some cases by acute urinary retention due to functional infravesical obstruction, in others – by hyper- and hyporeflective activity of the muscular apparatus of the bladder, including detrusor-sphincter dyssynergia (Goldman and Zimmern, 2006; Zhuravlev et al., 2013). At the same time, previous studies showed that compensated neurological disorders can occur under conditions of Dry Immersion (Gerstenbrand and Kozlovskaya, 1990; Berger et al., 2001).

In this regard, we can assume that the observed phenomena are of a functional nature due to support withdrawal and can be associated with both changes in psychological self-control and neurogenic changes at various levels of reflex circuits and arcs of the central and peripheral nervous system (de Groat, 1997; Danilov et al., 2016). Most likely the phenomenon of dysuria was reversible functional (Krivoborodov and Shkol‘nikov, 2004) due to support unloading and neurogenic changes at various levels of reflex circuits and arcs of the central and peripheral nervous system (de Groat, 1997; Danilov et al., 2016). We also do not exclude the possibility that this phenomenon may be associated with local neuromuscular disorders caused by changes in internal hydrostatic pressure and the rate of its increase on mechanoreceptors of the bladder walls due to the active redistribution of body fluids, as well as by changes in the sensitivity thresholds of mechanoreceptors associated with effects of microgravity. This may also be caused by disinhibition of previously compensated control of the detrusor sphincter apparatus of the bladder which has a pronounced individual character (Fry et al., 2010). In further studies, it would be interesting to check the effect of support unloading not only on skeletal muscles but also on smooth muscle organs (Kozlovskaya et al., 2007a,b; Shenkman et al., 2017; Shenkman and Kozlovskaya, 2019).

The negative effects noted here, on the one hand, carry some risks for the subjects, on the other hand, they confirm that Dry Immersion is close in its effects on the body to space flight and can be used, among other things, for urogenital and back pain syndrome studies.

### Comparison of Bed Rest and Dry Immersion Study

In contrast to research in real space flights, data on women’s HDBR are published more often, but not as often as we would like. Here we provide data from women’s HDBR and men’s DI that would be especially interesting to discuss.

Baseline HR and BP were comparable to those of women who participated in the 60-day HDBR (Guinet et al., 2009). After HDBR, the blood pressure of women remained unchanged, in contrast to our study, where a slight decrease in SBP (∼ 5 mm Hg) and DBP (∼ 3 mm Hg) was recorded. HR increased by 14 bpm and 18 bpm after 60-day HDBR and 3-day DI, respectively. After a short duration 7-day HDBR, no changes in pressure were also recorded, and the increase in HR was ∼ 10 bpm (Custaud et al., 2002). Systolic and diastolic blood pressure values in men were higher, both in the baseline and after 7 days of immersion (Borovik et al., 2020). In general, an increase in DBP is recorded for male immersions in the first week of immersion, in contrast to our study (Shul’zhenko et al., 1980; Borovik et al., 2020). Comparison of blood pressure and heart rate values (de Abreu et al., 2017; Amirova et al., 2020b) in DI of the same duration between men and women volunteers using two-way ANOVA showed a significant difference between the sexes in heart rate [*F*(1,80) = 94.57; *P* < 0.0001] throughout the entire observation period and in SBP [*F*(1,80) = 38.28; *P* < 0.0001] – only during immersion. The heart rate in women was, on average, 15 beats per minute higher, while SBP was lower by 14.8 mm Hg.

Cardiovascular changes are induced by fluid redistribution, which in turn affects water intake and diuresis. We were unable to find direct data on the volume of fluid secreted and consumed in the female HDBR, however, daily urinary excretion of urea was increased only in the female subjects. At the same time plasma volume changes were similar in both men (9.1 ± 1.4%) and women (9.4 ± 0.8%). Also, blunted increase of noradrenaline in women during the orthostatic test after 7-day HDBR was found, which is associated with their poorer orthostatic stability. Despite this fact, no sex differences in hydroelectrolytic changes have been found during HDBR (Millet et al., 2001).

In our study, the daily body temperature in most cases remained within the normal range (no more than 37.0), maintaining normal daily fluctuations during all the observation period. The decreased body temperature at the end of the first day of DI can be explained by the lower bath water temperature. With an increase in the water temperature on average for the group by 0.5°C, the body temperature returned to the baseline values. In men, the Immersion of the same duration had tended to increase body temperature by 0.2–0.3°C with rhythm disturbances after the end of the immersion (Amirova et al., 2016). The study of daily body temperature after 8 weeks of stay in HDBR conditions showed the preservation of oscillation period and amplitudes of circadian rhythms. However, a rhythm shift of 37.8 min over 29 days was found (Mendt et al., 2021).

As studies conducted earlier in men show, an increase in body height occurs in the first day of immersion, and then remains at this level throughout the entire exposure. Under conditions of 21-day DI, the average height in men increases by 2 cm (Tomilovskaya et al., 2020). In our study, women did not show a significant increase in height, which may be due to the greater plasticity of women’s connective tissue.

Also, it important to note that the time outside the immersion bath for women did not exceed that for men (Treffel et al., 2017; Amirova et al., 2020a). This is a good achievement as it is known that increasing the time outside the bath reduces the effects of DI (Navasiolava et al., 2011).

## Conclusion

The conducted study shows the possibility of the participation of women in the Dry Immersion model. On the one hand, the phenomenon of dysuria found in women during immersion requires additional supervision by medical personnel, and on the other hand, it confirms that the model reproduces space flight factors. Undoubtedly, studies of the female body under conditions of Dry Immersion require further study.

## Data Availability Statement

The raw data supporting the conclusions of this article will be made available by the authors, without undue reservation.

## Ethics Statement

The studies involving human participants were reviewed and approved by the Bioethical Commission of the Institute of Biomedical Problems of the Russian Academy of Sciences. The patients/participants provided their written informed consent to participate in this study.

## Author Contributions

ET and LA wrote the draft of the manuscript and made its revisions, they also played the key role in the organization of the experiment. IN, IR, and RC contributed to the part of medical support and medical risks of DI. SL prepared the part on the special device for urine collection including invention and manufacturing the device. AS prepared the part concerning the overall scheduling of the experiment and made the revision of the manuscript. IE and IP prepared the analysis of general data. IZ and TS prepared and revised the list and description of the studies. NS, VK, AR, VB, and NA contributed to the methods part and design of the figures. GV made the analysis of water balance. OO contributed in the global revision of the manuscript and was a supervisor of the experiment. All authors contributed to the article and approved the submitted version.

## Conflict of Interest

The authors declare that the research was conducted in the absence of any commercial or financial relationships that could be construed as a potential conflict of interest.
