# Supplementary figures and images for: The First Female Dry Immersion (NAIAD-2020): Design and Specifics of a 3-Day Study
Source: Front Physiol. 2021 Jun 14;12:661959. doi: 10.3389/fphys.2021.661959 (PMC8236811; doi:10.3389/fphys.2021.661959)

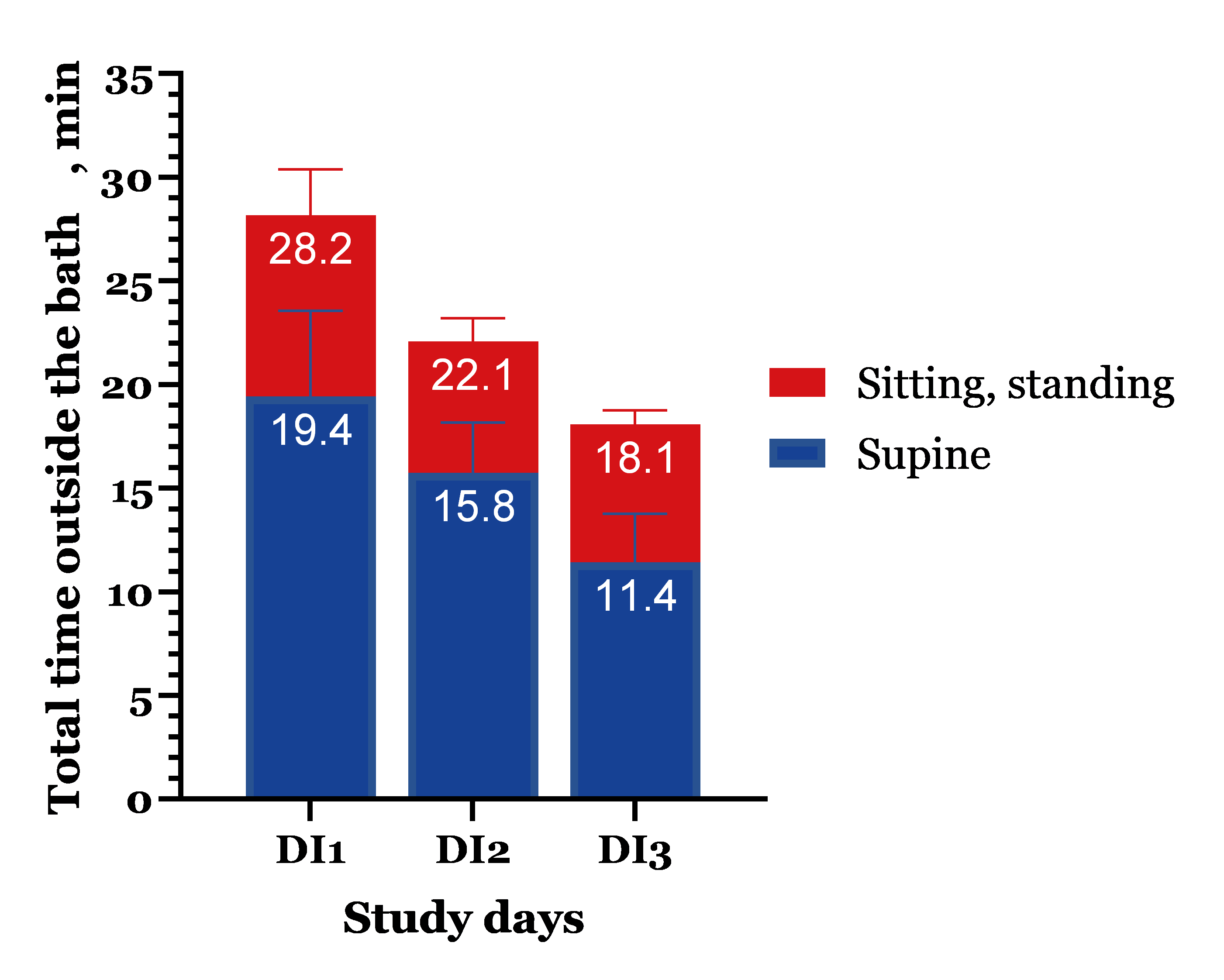

Supplement: Supplementary file 1 [file Image_1.tif]

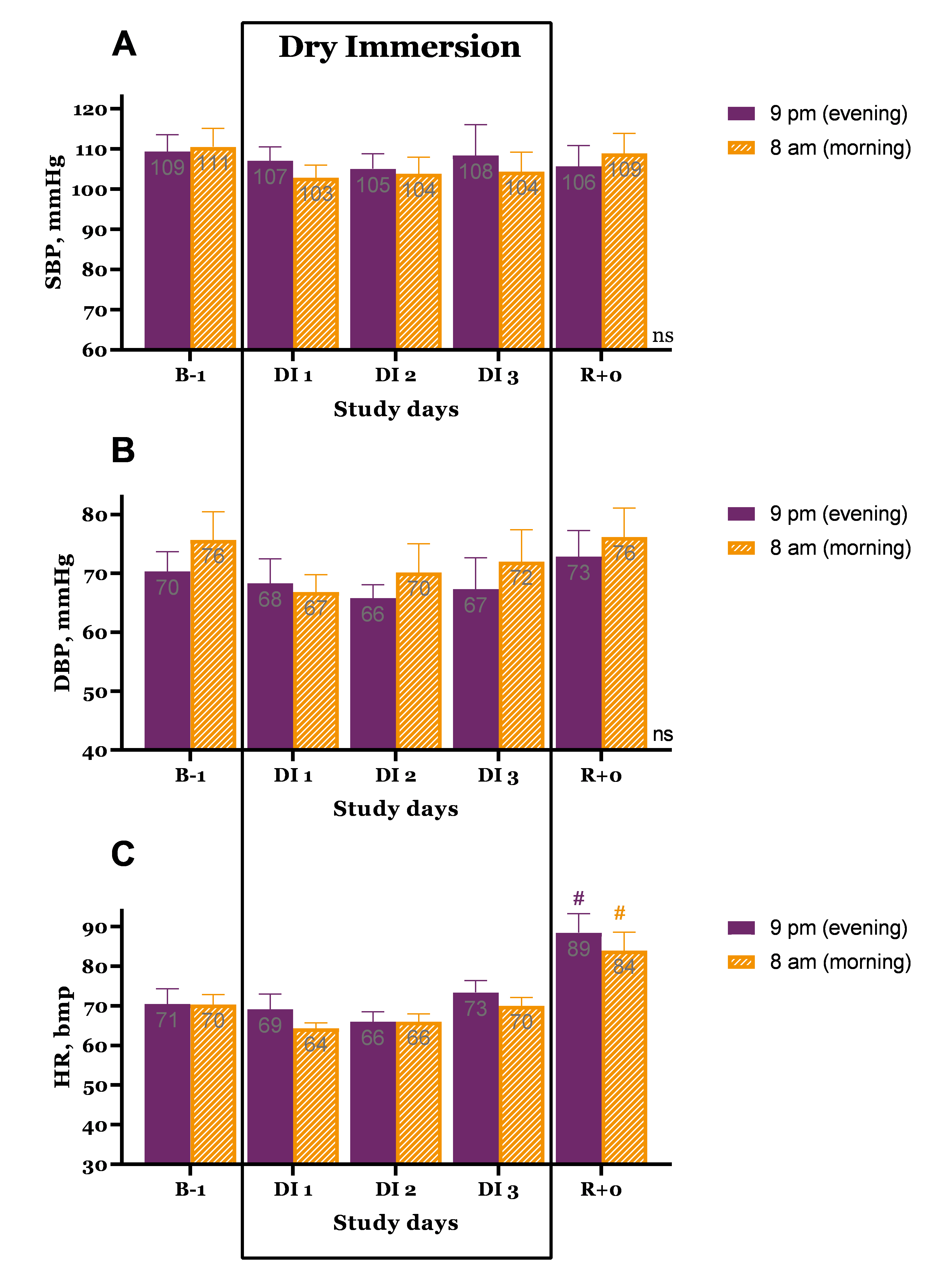

Supplement: Supplementary file 2 [file Image_2.tif]

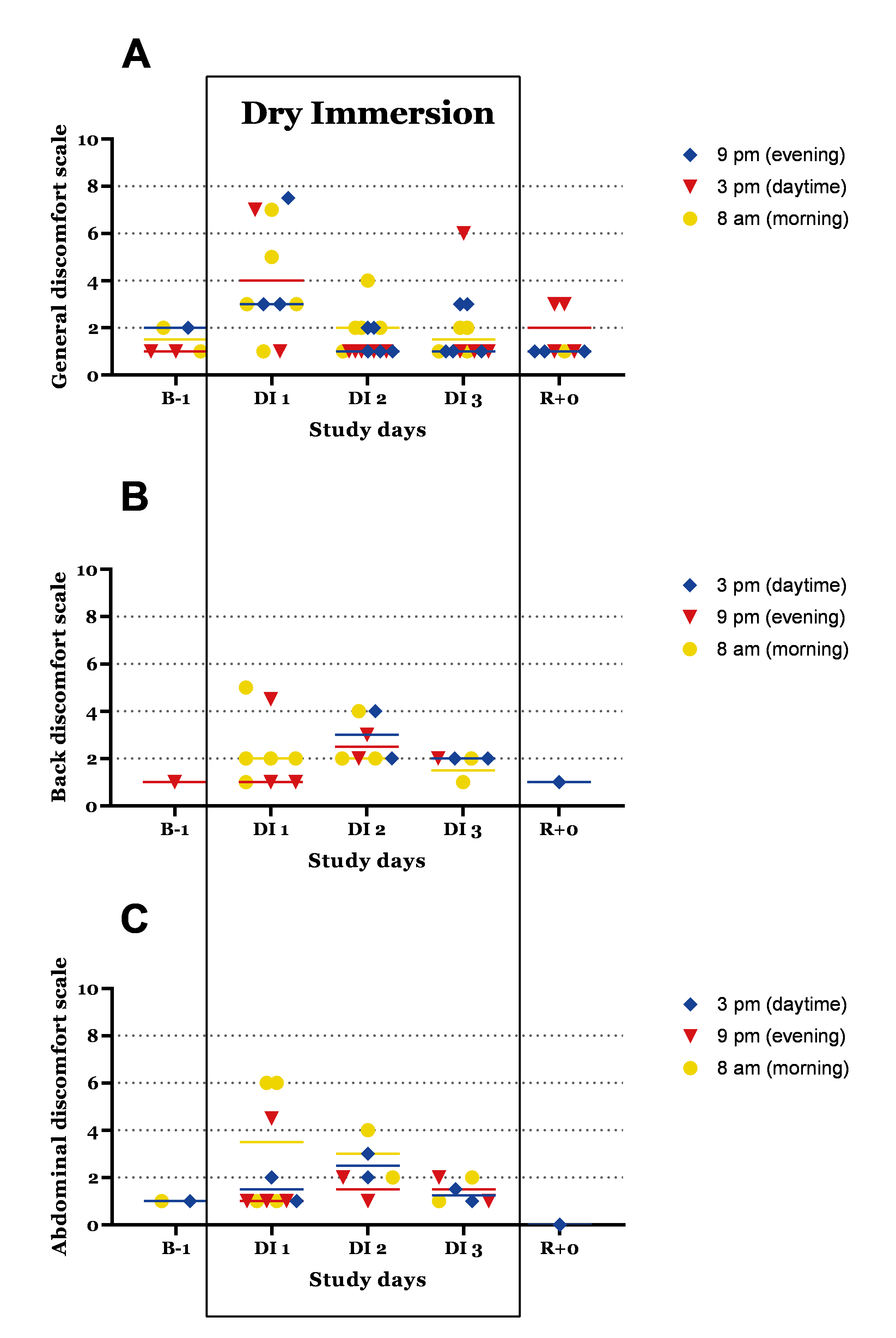

Supplement: Supplementary file 3 [file Image_3.tif]
