# Supplementary material for: The First Female Dry Immersion (NAIAD-2020): Design and Specifics of a 3-Day Study
Source: Front Physiol. 2021 Jun 14;12:661959. doi: 10.3389/fphys.2021.661959 (PMC8236811; doi:10.3389/fphys.2021.661959)
Supplement: Supplementary file 4 [file Table_1.docx]

**TABLE A** | Tests carried out during the experiment*

| Approaches and techniques used | Days before DI | Days in DI | Days after DI | Explanations |
| --- | --- | --- | --- | --- |
| Neurophysiological studies | | | | |
| 1. Stiffness of muscles, ligaments and tendons | -4, -3, -1 | daily | +0, +2 | Viscoelastic properties of muscles, ligaments and tendons of torso and limbs (non-invasive) |
| 2. Precision control of hands and feet | -4, -3 | 1, 3 | +0, +3 | Precision characteristics (minimum, maximum efforts, as well as minimum differentiable difference between efforts) of arm and leg movements |
| 3. Н-reflex | -5, -2 | 3 | +2 | Characteristics of H-reflex of mm. soleus and gastrocnemius |
| 4. Magnetic stimulation | -3, -1 |  | +0, +3 | Characteristics of motor responses of mm. soleus and gastrocnemius caused by transcranial and transspinal magnetic stimulation |
| 5. Vertical stability | -6, -4, -2 |  | +0, +1, +2, +3 | Postural stability and postural correctional responses caused by disturbances of various modality |
| 6. Maximum strength and endurance in high jump | -3 |  | +1 | Speed-power characteristics of muscles-extensors of lower limbs and assessment of endurance |
| Functional studies | | | | |
| 7. Electrocardiography and tachooscillography of the heart | -2 | daily | +2 | Study of changes in work of cardiovascular system associated with changes in blood plasma proteome |
| 8. Doppler flowmetry, hand capillaroscopy | -2 | 3 | +2 | Functional state of microcirculatory bed |
| 9. Bioimpedance analysis | -7 | 2 | +6 | Dynamics of body composition and redistribution of body fluids |
| 10. Computerized foot plantography | -1 | daily | +2 | Morphology of feet, tendons and ligaments |
| 11. Computerized eye perimetry and tonography | -7, -3 |  | +0, +6 | Study of effect of adaptive hypohydration of organism on state of intraocular hydrodynamics in correlation with aldosterone-progesterone balance |
| 12. Ultrasound study of triceps muscle of shin | -1 |  | +0 | Architecture of skeletal muscles - angle of inclination and length of fibers in heads of calf muscle |
| 13. Ultrasound angioscanning | -7 |  | +0, +6 | Visualization of venous and study of changes in venous hemodynamic parameters |
| 14. Ultrasound study of kidneys | -7 |  | +0, +6 | Visualization of kidneys, identification of possible changes, and study of changes in blood flow parameters |
| 15. X-ray osteodensitometry | -7 |  | +0, +6 | Bone status and body composition |
| Biological sample studies | | | | |
| 16. Biopsy of soleus muscle of shin | ~25-30 |  | +0 | 1. Study of key intracellular signaling systems in postural muscle of shin  2. Study of content of a large number of highly abundant proteins in soleus muscle of shin |
| 17. Venous blood | -7 |  | +0, +6 | Concentration of hormones in plasma and serum, indicators of water-electrolyte metabolism, markers of bone metabolism, renal function and state of cardiovascular system |
| 18. Capillary blood | -7, -2 | daily | +1, +2 | Plasma proteome characteristics and study of mechanisms of regulation of plasma levels of proteins that perform adaptogenic function |
| 19. Urine | -7 | daily | +0, +6 | General urine analysis and markers of bone metabolism and certain hormones |
| 20. Microflora samples | ~24-29, -1 |  | +0, +1 | Vaginal microflora and microflora of mucous membranes of upper respiratory tract, integumentary tissues and in intestinal microbiome |
| Psychophysiological studies | | | | |
| 21. Operator activity assessment | -2 | 1, 3 | +2 | Psychophysiological state of the subjects |
| 22. Movement monitoring | -3, -2, -1 | daily | +0, +1, +2 | Quality of sleep by comparative analysis of periods of night rest and physical activity (anxiety) identified by actigraphy during night |

* - The results of these studies are not presented in this article. The data will be published as they are processed and analyzed.
