# Supplementary material for: The First Female Dry Immersion (NAIAD-2020): Design and Specifics of a 3-Day Study
Source: Front Physiol. 2021 Jun 14;12:661959. doi: 10.3389/fphys.2021.661959 (PMC8236811; doi:10.3389/fphys.2021.661959)
Supplement: Supplementary file 5 [file Table_2.docx]

**TABLE B** | The volume of studies for purposes of medical control

| Measurements | Time of Day | Method | Statistical data processing method |
| --- | --- | --- | --- |
| Blood pressure | 15:00, 21:00, 8:00 | Tonometry at forearm level (according to Korotkov) with automatic tonometer | Two-way RM ANOVA (time of day × day) |
| Heart rate | 15:00, 21:00, 8:00 |  | Two-way RM ANOVA (time of day × day) |
| Body temperature | 15:00, 21:00, 8:00 | Thermometry in armpits with mercury thermometer | Due to two missing measurements, mixed-effects model (REML) was used (time of day × day) |
| Anthropometry | 21:00 | Measurement of height and weight in upright position with height meter and scales | Ordinary one-way ANOVA |
| Water balance | Daily | Counting of total volume of consumed liquids (including soups, tea, liquid dairy products) and discharged urine per day (9.00 am was taken as beginning of a day) | Ordinary one-way ANOVA |
| Questionnaires | 15:00, 21:00, 8:00 | General discomfort, pain in back and abdomen on a 10-point scale, where 1 is minimum sensation of pain/discomfort, 10 is maximum one | Not used |
| Examination and collection of anamneses | 15:00, 21:00, 8:00 | General medical examination, filling questionnaire for medical assessment of dynamics of physiological adaptation in order to identify and search for traits of organism's reaction | Not used |
